# Barriers and facilitators to the implementation of brief interventions targeting smoking, nutrition, and physical activity for indigenous populations: a narrative review

**DOI:** 10.1186/s12939-019-1059-2

**Published:** 2019-11-05

**Authors:** Mojan Fazelipour, Frances Cunningham

**Affiliations:** 1 Faculty of Pharmaceutical Sciences, University of British Columbia, 2405 Wesbrook Mall, Vancouver, BC V6T 1Z3 Canada; 2 Wellbeing and Preventable Chronic Disease Division, Menzies School of Health Research, Level 10, 410 Ann Street, Brisbane, QLD 4000 Australia

**Keywords:** Indigenous peoples, Early medical intervention, Biobehavioral sciences, Risk reduction behavior, Implementation science

## Abstract

**Objective:**

This narrative review aimed to identify and categorize the barriers and facilitators to the provision of brief intervention and behavioral change programs that target several risk behaviors among the Indigenous populations of Australia, Canada, and New Zealand.

**Methods:**

A systematic database search was conducted of six databases including PubMeD, Embase, CINAHL, HealthStar, PsycINFO, and Web of Science. Thematic analysis was utilized to analyze qualitative data extracted from the included studies, and a narrative approach was employed to synthesize the common themes that emerged. The quality of studies was assessed in accordance with the Joanna Briggs Institute’s guidelines and using the software SUMARI – The System for the Unified Management, Assessment and Review of Information.

**Results:**

Nine studies were included. The studies were classified at three intervention levels: (1) individual-based brief interventions, (2) family-based interventions, and (3) community-based-interventions. Across the studies, selection of the intervention level was associated with Indigenous priorities and preferences, and approaches with Indigenous collaboration were supported. Barriers and facilitators were grouped under four major categories representing the common themes: (1) characteristics of design, development, and delivery, (2) patient/provider relationship, (3) environmental factors, and (4) organizational capacity and workplace-related factors. Several sub-themes also emerged under the above-mentioned categories including level of intervention, Indigenous leadership and participation, cultural appropriateness, social and economic barriers, and design elements.

**Conclusion:**

To improve the effectiveness of multiple health behavior change interventions among Indigenous populations, collaborative approaches that target different intervention levels are beneficial. Further research to bridge the knowledge gap in this topic will help to improve the quality of preventive health strategies to achieve better outcomes at all levels, and will improve intervention implementation from development and delivery fidelity, to acceptability and sustainability.

## Background

Historical commonalities in colonization, assimilation, and ongoing socio-economic disadvantages have made the Indigenous populations of Australia, Canada, and New Zealand more vulnerable compared to their non-Indigenous counterparts, and they experience higher rates of chronic disease risk factors [1, 2]. From the range of health risk behaviors, poor nutrition, low levels of physical activity, smoking and obesity have been disproportionately higher in these population groups [1, 3–5]. These lifestyle risk factors have also been recognized by the World Health Organization (WHO) as being the major preventable causes of chronic diseases across the world [6]. The Australian Institute of Health and Welfare (AIHW) has identified chronic diseases (caused by modifiable risk factors) as the main factors responsible for the gap in mortality among the Aboriginal and Torres Strait Islander people in comparison with the general population [7].

Of significance, the clustering of such determinants tends to be more prominent in socioeconomically-disadvantaged groups, such as Indigenous populations, and the synergistic interaction of such risk factors has been shown to be responsible for detrimental health outcomes including elevated morbidity and mortality among these population groups [8, 9]. As such, it is critical to consider the smoking, nutrition, and physical activity (SNP) risk factors collectively rather than as individual entities. This is mainly due to the intersecting connections in the nature of such behaviors. In addition, interventions that address several behavioral risk factors have been shown to be more effective than single interventions in tackling the burden of chronic diseases [10]. It is, thus, believed that the adoption of a broader multiple-risk intervention approach should further facilitate the design and implementation of effective preventive or recovery strategies for Indigenous populations.

There is, indeed, growing evidence highlighting the effectiveness of multiple health behavior change (MHBC) interventions [11]. Among the primary prevention strategies, brief intervention (BI) programs, as the most cost-effective method to target multiple SNP risk factors, have been recognized as evidence-based initiatives for prevention and management of chronic health conditions [9, 12]. Previous studies have shown promising results from the use of brief motivational interventions as preventive health promotion strategies [13]. The targeting of MHBC is a fairly recently introduced approach and this is evident through the paucity of research in this area. Although there is a large body of evidence on the effectiveness of brief interventions and behavioral interventions, the pool of literature reduces significantly in relation to such interventions for Indigenous populations.

Therefore, identifying the barriers and enablers to the successful implementation of these beneficial preventive strategies in the three countries, sharing similarities in Indigenous history, health system governance, constitutional agreements, and recognition from the state or provincial governments [14, 15], should assist with improving their wider availability and their performance [8].

The primary objective of this narrative review was to identify the barriers and facilitators to implementing brief intervention and behavioral change programs that target several risk behaviors among the Indigenous populations of Australia, Canada, and New Zealand, and to portray the current state of knowledge on this topic In addition to the highlighted importance of such lifestyle behaviors in the prevention of chronic disease development, the major focus of this review on lifestyle risk factors was in line with the objective of the Queensland Health Aboriginal and Torres Strait Islander Brief Intervention Training Program (B.strong), developed by Menzies School of Health Research [16]. B.strong aims to build the capacity of Queensland’s community and primary health workers to deliver smoking, nutrition, and physical activity brief interventions to Aboriginal and Torres Strait Islander people [16]. Exploring the current state of knowledge on this topic, and highlighting any knowledge gap in this area will help in directing future research to improve the quality of intervention implementation of health promotion strategies from program development and delivery fidelity, through to acceptability and sustainability.

## Methods

The review was undertaken in accordance with the **P**referred **R**eporting **I**tems for **S**ystematic reviews and **M**eta-**A**nalysis (PRISMA) guidelines.

### Search strategy

With the assistance of a librarian, six electronic databases were searched: PubMed, Embase, CINAHL, HealthStar, PsycINFO, and Web of Science. The search consisted of keywords and subject headings, the details of which are included in an Additional file 1. In the initial development of the search strategy, the search terms were grouped into three conceptual categories according to the Population Intervention Setting/Comparison Outcome (PISCO) analysis framework: (1) brief intervention (i.e. brief intervention*, brief advice, brief intervention training program, Screening, Brief Intervention, and Referral to Treatment (SBIRT), (2) areas of risk: Poor nutrition, physical activity, and smoking (i.e. Nutrition* OR Diet OR Food* OR Dietary OR Smoking OR tobacco OR cigarette* OR Physical activit* OR exercise* OR physical fitness OR obesity OR obese OR overweight OR diabetes), (3) Indigenous population (i.e. Indigenous OR Aborigin* or first nations OR inuit* OR metis OR native OR indian OR aboriginal and Torres strait islander OR maori OR eskimo*). Several trial searches were conducted and as the study aimed at retrieving and reviewing interventions targeting multiple risk factors, it was decided to employ a broader search strategy using the two main conceptual categories - (1) brief intervention and (3) Indigenous population. Then, a thorough review of relevant results was conducted to capture published peer-reviewed journals meeting the inclusion criteria. Details of the database search as well as the terms utilized are provided in a separate file (Additional file 1).

### Identification, screening, quality assessment, and inclusion of publications

Conduct of the search across the six databases retrieved a total of 741 citations.

A total of 75 peer-reviewed journal articles was selected following the initial abstract and title screening. An additional hand search of bibliographies of articles retrieved from the search was carried out to identify other relevant publications on the subject (*n* = 2). Studies were included if (1) they were published in peer-reviewed journals from 2007 to 2018; (2) they discussed at least one facilitator or barrier in terms of brief intervention implementations that addressed SNP risk behaviours; and (3) they included Indigenous peoples in Australia, Canada, or New Zealand. To ensure rigor and relevance, the selection of interventions was guided by the definition of brief intervention by Nilsen et al. where ‘brief intervention’ encompasses any preventive, patient-focused, and motivational counselling that is performed by healthcare professionals in short timespans, aimed at changing behavioral risk behaviours [17].

Following a more robust abstract review, articles were excluded if: (1) the study population was not exclusive to Indigenous peoples of any of the three countries; or (2) they did not present MHBC programs (i.e., they only focused on one risk factor).

The search identified a large gap in brief intervention studies in Canada and New Zealand, although the same definition of brief intervention is used in these countries. Following consultation with Indigenous health researchers in both Canada and New Zealand, two common explanatory themes emerged based on their expert opinion and on our thorough thematic analysis of the selected studies: (1) although many similar programs may run across countries with Indigenous peoples, there is a large gap in academia in terms of research and publication of peer-reviewed evaluations of such interventions; (2) brief intervention programs in Canada appear to be mostly focused on substance abuse and alcohol management programs.

Thus, to include relevant findings from those countries, and to address the scarcity of such programs in terms of the type of intervention in Canada and New Zealand, the search also included studies that focused on community-based and/or family-based interventions that met the same inclusion criteria, if the components of the programs were similar to those for brief interventions. Importantly, this decision drew upon the findings of a study highlighting the effectiveness of community-level strategies in targeting SNP risk factors in comparison with individual-level programs among Indigenous populations [18].

**Fig. 1:**
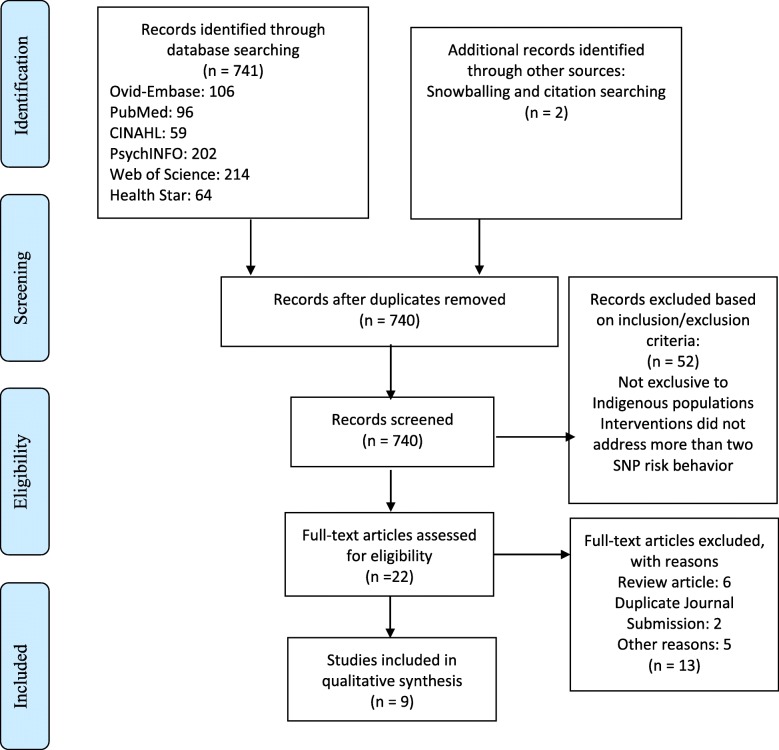
PRISMA flow chart

Overall, 22 articles met the criteria for full text review. A sensitive and comprehensive review was conducted of these articles by the authors to ensure their rigor and relevance in meeting our review criteria, and this yielded nine articles for the review (Fig. 1). Studies were excluded if they were review articles, presented duplicate findings of journal publications, or reported on the same program.

The quality of studies was appraised in accordance with the Joanna Briggs Institute (JBI) guidelines, and by utilizing the JBI System for the Unified Management, Assessment and Review of Information (SUMARI) [19].

### Data synthesis and presentation

Thematic analysis was used to synthesize findings and a narrative approach was used to present findings in accordance with the JBI guidelines [20]. To enhance methodological rigor, transparency, and reproducibility, a PRISMA checklist (Additional file 2) was completed and a flow chart was produced (Fig. 1).

## Results

### Description of included studies

Table 1 provides a summary of study characteristics including intervention designs, methods, and level of intervention of the articles retrieved from the search. Overall, of the nine articles included for the final review, three Australian studies were included, two of which focused on the evaluation of MHBC SNP brief interventions that targeted Indigenous Australians [9, 12]. The other Australian article, a cross-sectional study, analyzed the preferences and priorities of Indigenous populations with different aspects of MHBC [8]. Three articles from New Zealand were included: these studies evaluated the impact of community-based lifestyle behavior interventions on lifestyle risk factors among Maori populations [21–23]. Finally, three Canadian studies were included. One study focused on the evaluation of a school-based behavioral intervention to improve physical activity and healthy eating in three remote First Nations communities in Canada based on a community-based participatory research approach [24]. The other two Canadian studies focused on the evaluation of healthy lifestyle interventions in Aboriginal communities in Canada [25, 26].

**Table 1:** Characteristics of included studies

Citation^a^	Author (year)	Country	Design	MHBC(s) targeted	Type of interventions(s)
[8]	Noble et al. (2016)	Australia	Observational/Cross-sectional	SNP	Community-based
[9]	Clifford et al. (2010)	Australia	Mixed methods	SNP	Individual
[12]	Panaretto et al. (2010)	Australia	Mixed methods	SNP	Individual
[21]	Simmons et al. (2008)	New Zealand	Experimental/Randomized cluster controlled trial	Nutrition and Physical activity	Community-based
[22]	Coppell et al. (2009)	New Zealand	Descriptive/Process evaluation of findings reported via a case-study approach	Nutrition and Physical activity	Community-based
[23]	Hamerton et al. (2012)	New Zealand	Descriptive/Process evaluation of findings reported via a case-study approach	Nutrition and Physical activity	Community-based
[24]	Tomlin et al. (2012)	Canada	Pre-experimental	Nutrition and Physical activity	Community-based
[25]	Anand et al. (2007)	Canada	Experimental/Randomized open trial	Nutrition and Physical activity	Family-based
[26]	Mead et al. (2012)	Canada	Semi-experimental/Quasi-experimental pre-and post-evaluation	Nutrition and Physical activity	Community-based

^a^Number under citation refer to the corresponding numbers in the reference list

### Study characteristics, findings, and level of intervention

Studies were categorized by the intervention level reported on in the study. Based on the characteristics of the included studies, the three intervention levels were identified as (1) individually-based brief interventions, (2) family-based, and (3) community-based behavioral interventions which targeted more than one of the chronic disease risk factors of smoking, nutrition, and physical activity. The priorities and preferences of Indigenous populations with regard to MHBC strategies were also presented in a separate category as they could be applied to all three intervention levels while also providing insights into the analysis of the barriers and enablers.

### Individual-level brief interventions

One study used a mixed-methods approach to evaluate the performance and organizational capacity of health services in delivering brief interventions on smoking, nutrition, alcohol, and physical activity (SNAP) risk factors across four Aboriginal and Torres Strait Islander medical services in Queensland [12]. Study methods involved the conduct of surveys and focus groups in addition to medical chart reviews to assess knowledge and to qualitatively analyze the barriers and facilitators associated with the available brief intervention programs. The authors identified several barriers by collecting feedback from 46 respondents out of 50 clinical staff (92% response rate) who participated in the study [12].

This study found significant inconsistencies and poor quality in the recording of SNAP risk factors and their assessment measures in medical charts [12]. In addition, the electronic medical records (EMRs) lacked a field pertaining to nutritional assessment information. Another major barrier discussed by the authors was the practitioner’s perception of time constraints providing a barrier to establishing a good relationship with the patient. The study found there was a longer time associated with the recording of nutritional and physical activity data (almost two times longer than for tobacco and alcohol screening). Socioeconomic barriers seemed to play a major role in relation to client attendance for follow-up clinical visits as well as in the affordability of recommended lifestyle options (e.g. healthy foods) [12]. Furthermore, both the high turnover of staff and the high-risk lifestyle behaviors of the staff themselves provided major obstacles to successful brief intervention implementation. Importantly, the study noted that the lack of confidence of staff and their perceptions of the sensitivity of discussing lifestyle risk behaviors with their clients could challenge the performance of brief interventions, especially when the risky behaviors have been normalized in the communities [12].

Another study reviewed brief intervention resource kits targeting SNAP risk factors for Indigenous Australians to assess the content and quality of such resources [9]. The study method included identifying phone contacts, the conduct of surveys, and the review of the resource guides [9]. Overall, 15 kits were identified from the 74 organizations contacted. The format, elements, information contents, and readability of client resources were assessed according to several clinical and health promotion guidelines [9]. Among the identified resource kits, only one brief intervention program addressed nutritional and physical activity behavioral risk factors. The major components missing from the resource kits were evidence-based guidelines, screening, the means of decision making and training resources [9]. The authors noted that these findings may indicate an absence of expertise and support in Indigenous communities in their production of such resource materials. Materials included in the packages differed with regard to the behavior change models and risk measurement tools. The authors suggested that the findings might be an indicator of different objectives relating to the specific population of focus for the development of the brief intervention kits. Information packages for clients also seemed to be missing from some of the resources [9].

### Family-based behavioral interventions

Canadian research found that family-based collaboration models were effective in improving the health of an individual within a group (a family) [23]. This is likely to be associated with the importance of the family unit in Aboriginal culture and in societal values, and in recognition of this, the design of such programs is based on family support and strengths [23]. This approach was utilized to design a family-based intervention targeting nutrition and physical activity targeting Six Nations Reserve families in Brant County, Ontario. This intervention took place over a period of 6 months among 29 family units [25]. In this randomized community intervention, through regular household visits over the period of the study, Aboriginal health counsellors were responsible for assisting families in setting dietary and physical activity goals. They also assessed primary and secondary outcomes of such behavior changes, including the change in dietary intakes and physical activity, to longer-term impacts such as weight loss and body fat reduction. The intervention included distribution of filtered water and educational programs for family members. The results of this study revealed improved physical activity and dietary habits measured after 6 months compared with baseline data. However, the change in patterns of such behaviors was not statistically significant. The major barriers identified by the Six Nations Health Committee were structural barriers such as: poor walkability of neighborhoods, lack of bicycle paths, safety concerns, and scarcity of natural and fresh food products. On Reserves, fresh fruit and vegetables are relatively expensive and they have limited shelf-life [25]. Overall, the effectiveness of the intervention was likely adversely influenced by the social disadvantage faced by Aboriginal families living on the Reserve. Such structural barriers for the community could not be overcome simply by advice to communities to alter their food choices and activity patterns.

### Community-based programs

In New Zealand, a health promotion program called REPLACE was developed to improve healthy lifestyles of Maori populations. Hamerton et al. [23], presented the results of the program evaluation. This program, which was a part of a larger initiative called ‘Healthy Eating Healthy Action’ (HEHA), was delivered through six community-based Maori health agencies that identified their particular needs and priorities within the local community. This health promotion initiative supported and encouraged substituting every modifiable lifestyle behavior with a healthy alternative in the areas of exercise, nutrition, smoking, and alcohol consumption over a two-year period (2007–2009). Taking into account the four dimensions model of Maori health (body, mind, spiritual, and family), the program focused on facilitating multiple behavior changes through supportive environments in each of the six regional health agencies, based on their unique set of priorities. The program components included, for nutrition: nutritional education, cooking sessions, health agency policy change, and community gardens to promote healthy eating; and for physical activity: cultural dance, exercise classes, and providing fitness equipment to target physical activity; or a combination of both initiatives such as gathering fruit.

The approach recognized the large role small lifestyle changes could play in people’s quality of life over time. Process and short-term outcome evaluation of the intervention showed successful changes at individual, family, and community levels. The evaluation method fostered a face-to-face approach among participants, stakeholders, and project coordinators, and qualitative data were collected over a two-year period by means of participant observation, surveys and interviews, holding focus groups, and monthly staff meeting reports [23]. The major enablers of the program’s effectiveness were identified as: (1) the innovative approaches of each community in implementing the program based on their unique set of needs and appreciating their distinct cultural values; (2) the ability of the health promotion program to respond to changing needs over time both in terms of environmental factors and clientele; and (3) the leadership and participation of Maori individuals in the program implementation that led to enhanced acceptability of healthy messages by participants using a ‘Maori reaching Maori’ approach [23].

The Vanguard study was a pilot study of a cohort of 160 Maori participants in the New Zealand Maori Community Health Worker (MCHW) intervention, Data collection was conducted both prior to and during the MCHW intervention. The major study was aimed at improving lifestyle risk factors for diabetes [21]. The intervention, delivered by MCHWs, was based on the notion that the degree to which a person requires health worker support throughout the intervention might be reduced if he/she addresses lifestyle behavioral changes within the family or community. This randomized cluster-controlled trial study for lifestyle modification was a combination of a community, family, and individual level program to promote lifestyle changes based on the baseline Maori physical activity and nutritional behaviours [21]. The training kits for MCHWs included biological health modules, motivational training, communication skills, and details and background knowledge regarding 12 key lifestyle change messages covering nutrition and physical activity.

The results of the pilot study conducted on the initial study participants (*n* = 160) showed a significant weight reduction for participants over the course of the intervention (189 days), compared with baseline data. Several facilitators contributed to the high acceptability of this program among the Indigenous population. First, the intervention was promoted through media, cultural events, and by the majority of the community members. The major focus in intervention delivery was on building trust and respecting family and community relationships. The core of the intervention was based on the key ‘message’ approach - created to set small success points to further improve perceived control over multiple lifestyle changes. Also, the design of the study included determining barriers and solutions to facilitate such changes. The promotion of preferred types of physical activities (identified by the community) was another enabler for the observed success rates of the program. However, conflicts existed in terms of participants’ dietary preferences (higher fat and protein than recommended in guidelines) and their macronutrient components (fat, protein, and carbohydrate) [21].

Ngati and Healthy intervention in New Zealand was a community-led diabetes prevention healthy lifestyle intervention program aimed at modifying high risk behavioral factors in areas of physical activity and nutrition: promoting weight loss, increasing exercise and adoption of healthy eating habits over a period of 2 years [22]. The major components of the program included health promotion, and educating individuals across the community who were at higher risk, adopting a collaborative approach that involved local schools, businesses, and organizations. Early engagement of community health workers in the project facilitated the sustainability and acceptability of the program throughout the population’s routine daily activities. Two surveys were conducted (of 286 out of 741 eligible and 235 out of 701 eligible participants respectively) over the two-year intervention. The process evaluation of the intervention highlighted insightful outcomes such as the increased participation of young mothers and women in the program activities. The latter was seen as a valuable outcome considering the important role of mothers and young women in family lifestyle practices. Data were assessed based on gender and on two age groups (25–49 and 50+ years) Overall, the project results showed a significant decrease in the risk of developing diabetes by reduced insulin resistance prevalence, and increased physical activity rates over the period of 2 years. This change was most significant for the group consisting of women between the ages of 25–49 years of age [22].

A Canadian study evaluated the effectiveness of a school-based intervention (Actions Schools! BC) targeting physical activity and nutrition among Indigenous children and youth in three remote Indigenous communities of British Columbia (Canada) [24]. Employing a case-study design, the intervention was planned across six delivery zones: (1) school setting, (2) physical activity sessions, (3) classroom activities, (4) families and communities (5) extracurricular, and (6) school spirit. The program, delivered over 7 months, facilitated delivery of an individual-based intervention to improve healthy eating habits and physical inactivity by participants including teachers and school staff, and individually-based action plans were delivered to 148 children and youths. The intervention employed family and community components with an emphasis on collaborative approaches in promoting healthy living [24]. The focus on community involvement and control over behavior change strategies were some of the major strengths of this intervention [24]. Overall, the program demonstrated no evidence of improvement in healthy eating and physical activity, measured by accelerometry and self-report both at baseline and post-intervention. However, health promotion programs targeting individuals early in life are more likely to have longer term benefits which were not measured by this study [24, 27].

Healthy Foods North (HFN) was a community-based, multi-institutional intervention to promote healthy lifestyles among the Indigenous populations of the Canadian Arctic (four interventions and two comparison groups in Nunavut and Northwest Territories) [26]. This program of 1 year’s duration aimed to address the psychosocial factors associated with nutrition and physical activity in four communities. The study was developed according to the constructs of the social cognitive theory and psychosocial models, using formative research and community participatory research methods. The major elements of the study included environmental, collaborative, and educational aspects. Outcome measures included psychosocial constructs, food-related behaviors: occurrence rate of food obtainment and food preparation techniques based on healthiness level, and body mass index (BMI). Evaluation assessment data were collected from a total of 246 individuals in the intervention and 133 in comparison groups. Based on data gathered pre- and post- intervention using the Adult Impact Questionnaire (AIQ), there was a significant improvement in the outcome measures pertaining to psychological and social constructs measuring knowledge of healthy eating habits, self-efficacy, and behavioral change intention. These changes were more prominent in overweight, obese and high-risk individuals with higher socioeconomic status (SES). Overall, the study highlighted the effectiveness of using culturally sensitive, community-based interventions that focused on capacity building and community partnership in improving healthy behavior change psychosocial factors to reduce chronic disease risk [26]. Nevertheless, the study found that to observe long-term behavior change outcomes more emphasis was needed on sustainability, improved implementation, and the evaluation also needed to take into account SES and health status differences among individuals [26].

### Priorities and preferences of indigenous populations in MHBC interventions

A recent Australian cross-sectional study was conducted to identify the determinants of acceptance of MHBC programs for an Indigenous population in Australia. The study had three main objectives: (1) assessing readiness towards high risk lifestyle changes; (2) acceptability of types of MHBC models: synchronously, in a sequence, or separately; (3) preferences for types of support programs, and the socioeconomic determinants responsible for the preferred choices [8]. A total of 211 participants, clients attending an Aboriginal Community Controlled Health Service in New South Wales, completed an anonymous questionnaire prior to their visits at the health center.

Survey results showed that on the client’s readiness to change their lifestyle health behaviors across all risk factors, smoking was the participants’ number one choice for behavior change [8]. Also, the number of risk factors seemed to directly correlate with the patients’ readiness to change (determined according to the stages of change model constructs) [8]. When participants were surveyed about the MHBC acceptability, among those in the contemplation phase of behavior change, only 32% preferred making changes synchronously. The highest percentage, 44%, indicated that they preferred making single changes sequentially and independently from one another [8].

Those who indicated willingness to change at least a single high-risk behavior had a preference for receiving healthcare practitioner help over other types of support, and, in particular, for improving nutrition and physical activity [8]. Receiving individual help and advice was also determined by 62% of clients as their preferred method of lifestyle behavior change support. In brief, the authors concluded that although MHBC was indicated to be acceptable by a population attending an Aboriginal Community Controlled Health Service, several factors including allowing flexibility in determining the risk behavior, readiness to change, the type of support demanded, and their delivery form, played an important role in the MHBC intervention’s success and acceptability [8].

Overall, the findings presented from the reviewed studies highlight the major facilitators and barriers to the implementation of brief interventions that address more than one modifiable risk behavior for chronic disease. Following a thorough thematic analysis, and due to the presence of varying outcome measures for both the quantitative and qualitative data extracted from the studies, the synthesized findings were grouped into four major categories based on the common themes that emerged: (1) characteristics of design, development, and delivery; (2) patient/provider relationship; (3) environmental factors; and (4) organizational capacity and workplace-related factors. To ensure consistency and relevance, the discussion of each theme and sub-theme is supported by data synthesized from the primary studies and their corresponding interventions.

## Discussion

### Characteristics of design, development, and delivery

#### Level of intervention and target for MHBC

To date, in targeting multiple lifestyle modifications, evidence has supported the superiority of behavioral and cognitive methods along with lifestyle counselling over other types of interventions [25]. Some research has shown that community-based interventions are significantly more effective compared to individual programs in terms of addressing lifestyle risk factors of obesity [23, 25]. However, other research in the past several years has reported that such interventions have not been effective in changing the multiple risk factors of obesity [25, 28]. The findings from a study on the effectiveness of brief interventions in changing nutrition behavior underscored the insufficiency of this method in dietary-related behavior change [29]. This could suggest that a collaborative and multi-level system approach is needed to tackle behavioral risk factors. The evaluation of the HFN program among the Indigenous peoples of the Canadian Arctic found that a community-based strategy that adopted several levels was an effective approach in targeting modifiable lifestyle risk factors in decreasing chronic disease prevalence [26]. As the high rate of chronic diseases among Indigenous populations is a complex issue shaped by myriad factors, multidimensional interventions should be further investigated in terms of their effectiveness in addressing the unique needs of these populations. Thus, in planning, implementing, and evaluating health promotion programs that target chronic disease risk factors, attention must be paid to complex factors associated with Indigenous communities (i.e. infrastructure, community capacity, and policies in place); as these community-level factors are essential to enhancing individual-level outcomes [30].

Priorities and preferences of Indigenous peoples must also be taken into account when considering the target level for a health behavior change intervention. For example, a cross-sectional study that assessed priorities and preferences of Indigenous populations on multiple behavior change found that when Indigenous clients were surveyed about their preferences over the level of support, the majority preferred individual-level supports for SNP health behaviors over family or community level approaches [8].

To analyze the evaluations of design and developmental characteristics of SNAP behavioral interventions among the Indigenous Australian communities, and to assess the interventions’ effectiveness in improving such risk factors, Clifford et al. [18], conducted a methodological review of 20 intervention studies. They found that one of the major barriers to effective replication and larger-scale delivery of behavioral interventions by other Indigenous community groups was the low quality and inconsistency of the reporting of methods and designs. The authors identified another challenge with analyzing the effectiveness of an intervention: the lack of a standard reliability and validity measure to quantify the outcomes.

#### Indigenous leadership and participation

Involving communities in the design and planning as pre-implementation steps in developing interventions is a critical factor in ensuring successful delivery and uptake of health promotion programs among Indigenous populations [31]. More specifically, Indigenous leadership and engagement, elements of intervention credibility, can further facilitate the implementation of behavioral programs [18, 28]. The ‘bottom-up’ approach in health promotion in which community members are involved in the design and implementation of programs by identifying their priorities can be more effective than ‘top-down’ strategies that are dictated by health authorities to the community [23]. For example, the delivery of the REPLACE project, which was implemented by a Maori project team which had strong connections with community members, was a strong determinant of the program’s acceptability by the population. Changes were not forced by ‘outsiders’ onto the community members, and the approach instead facilitated the community’s prioritized and identified needs [23]. Also, based on the theory of planned behavior as a model of behavior change, through empowering individuals, these community-led programs can potentially increase the perceived control of individuals over their high-risk behaviors and thereby facilitate behavior change [32]. In addition, engaging Indigenous community members in both pre-implementation and implementation processes may ameliorate their potential concerns about losing autonomy over their health.

The Ngati and Healthy intervention, a community-led project which was based on a community development approach and on the collaboration of community health workers and researchers, relied heavily on Indigenous participation and leadership in all phases of the project, including the design and development phase, delivery and implementation [22]. The authors reported that although community participation was time-consuming and demanding, it was an essential factor in ensuring improved feasibility, adoptability, fidelity, and sustainability of the lifestyle interventions for Indigenous populations [22]. Indigenous engagement in health research is an emphasized ethical guideline that improves practicality of implementation while it facilitates understanding of behavior changes [18].

#### Cultural appropriateness

Health promotion programs that are designed and implemented based on Indigenous cultural values have demonstrated higher perceived value among these population groups in targeting behavior change [23]. In addition, such programs are more likely to be sustainable over time as they empower individuals whose communities may have been subject to colonization and assimilation policies [23]. In the evaluation of the REPLACE project, the importance of cultural components in the sustainability of programs was emphasized [23]. Indeed, the Maori elders who participated in the project were constantly involved in passing on cultural practices to the younger generation to promote healthy lifestyle behaviors (e.g. collecting seafood).

In the Vanguard study, the development of intervention constructs based on Maori cultural values, both from the past and the present, was a major contributing factor to the high rate of program acceptability by Maori individuals [21]. Further, a Canadian review found that cultural elements that facilitated the delivery and adoption of lifestyle changes included use of traditional foods, focusing on the importance of the family in practicing lifestyle behavior role modelling, and assigning local family therapists for the delivery of the program [28].

#### Design elements

The quality of design components plays a critical role in enhancing successful implementation [9]. Based on their review of Indigenous brief intervention kits targeting chronic disease factors, Clifford et al. highlighted the importance of designing brief interventions using evidence-based strategies, along with consistent and standard validation measures. They also emphasized the importance of using written patient educational materials to strengthen and enhance verbal messages from the healthcare workers [9].

### Patient/provider relationship

Allocating enough time in primary care visits for health staff to build a relationship with clients can facilitate the delivery of lifestyle brief interventions, and can enhance the assessment of chronic disease risk factors [12]. Both relationship building and having regular visits with a client, are determinants of successful preventive interventions [9, 12]. The primary care setting is also well suited for the delivery of BIs as it provides first point of contact care to clients overtime [12]. Overall, evidence shows that establishing a good relationship built upon trust between the recipient and provider of lifestyle interventions is a major facilitator for implementing health behavior changes [31].

### Environmental factors

#### Social and economic barriers

In the review of five Canadian community-based interventions for Aboriginal populations including First Nations, Inuits, and Metis, there was no evidence of improved healthy weight outcomes [28]. Although several evaluations reported improvements in knowledge and beliefs regarding healthy behaviors, none of these programs showed effectiveness for physical activity and dietary intake outcomes. The major barrier discussed in this context was the broader social and economic determinants of health that could adversely impact on the effectiveness of such local multiple behavior change programs. These negative impacts are more concerning when it comes to lifestyle behaviors of Indigenous children as they have no direct control over such broader factors. In the healthy weight interventions, the programs showed that in spite of improved client knowledge and attitudes towards healthy lifestyle practices, there were socioeconomic barriers to achieving sustained positive lifestyle changes [28].

In the HFN project, the results of the study showed that the intervention had a higher impact on healthy eating and behaviors in higher socioeconomic status populations compared with their lower socioeconomic status counterparts [26]. The authors also emphasized the important role of socioeconomic status on adoption of healthy eating. Taking into account the interplay of social and economic barriers in addition to the unique characteristics of populations is critical in designing and implementing health promotion initiatives. Thus, in order to enhance and facilitate behavioral changes, preventive health programs also need to target the social and economic factors in Indigenous communities that provide barriers to the adoption of healthy behaviours [29].

### Organizational capacity and workplace-related factors

In assessing the organizational capacity of health services to deliver brief interventions for chronic disease risk factors to Indigenous Australians, Panaretto et al. identified several enablers and barriers to the successful delivery of health messages [12]. Enablers included effective communication and persistent leadership, appropriate and intensive staff training, team-based approaches in client care, developing consistent and standard EMRs by clinical staff, and providing Adult Health Checks (AHCs) funded by Australia’s universal health care system (Medicare) [12]. The barriers, on the other hand, were identified as high staff turnover rates, clinical workers’ own health status in addition to their lifestyle behaviors, and inconsistencies in utilizing various types of medical records [12].

Overall, these authors suggest that the barriers could be addressed to a reasonable extent through the development of assessment tools for modifiable risk factors, specifically nutrition and physical activity, along with appropriate training of staff in the delivery of the most recent state of knowledge that is evidence-based on SNAP risk factors, and by adding Adult Health Checks into the routine delivery of care.

A process evaluation found that barriers to the implementation of Actions schools! BC included high staff turnover, limited staff knowledge of healthy lifestyle behaviors, and minimal variation in choices. However, the ease of implementation and participant support were potential facilitators of such programs [33].

### Limitations of the review and future work

Despite employing a thorough and systematically-based method in searching databases, it is possible that this review may not have located all relevant Indigenous health promotion brief interventions from Australia, Canada, and New Zealand. The main purpose of this review was to identify the barriers and facilitators to the implementation of brief interventions that target MHBC in areas of smoking, nutrition, and physical activity among the Indigenous populations of Australia, Canada, and New Zealand. Unfortunately, varying outcome measures in behavioral interventions across the different countries limited any higher level or meta-comparison of such interventions. The level of the intervention also varied across different studies, largely due to the inclusion of community- and family-based interventions in addition to behavioral brief interventions.

We note that the specific inclusion criteria employed in this review made the limited pool of studies narrower. Firstly, there was a gap in the literature with regards to Indigenous-specific behavioral interventions. Secondly, the fact that the review looked for interventions that targeted multiple risk factors contributed significantly to the narrow body of literature. Thirdly, no brief intervention study targeting nutrition and physical activity was identified in either Canada or New Zealand (to the knowledge of the authors and the experts contacted). All retrieved brief intervention studies in Canada and New Zealand related to addressing substance abuse and addiction (including alcohol). Lastly, the exclusion of grey literature may have significantly limited the retrieved results since many evaluations of health promotion programs and projects are not published in the peer-reviewed literature. This was a common theme suggested by Indigenous health experts contacted throughout the review.

In conducting this research, we found a significant gap in the literature for systematic reviews analyzing multiple health behavior change interventions among vulnerable populations. The findings of this review make a contribution to the evidence relating to Indigenous populations in Australia, New Zealand and Canada. Further research is required to evaluate the effectiveness of brief and behavioral interventions for multiple health behavior change for Indigenous populations at different intervention levels. Findings of such research will assist in the development of recommendations and guidelines to inform policy changes and program funding support, and should facilitate the process of effective brief intervention program design, implementation, delivery, and evaluation.

## Conclusion

This review aimed to identify the enablers and barriers to the implementation of MHBC interventions among Indigenous populations drawing upon evidence from three countries. The high prevalence of chronic disease risk behaviors among the Indigenous populations has stemmed from the interplay of numerous underlying causes including the unique histories of population groups and the range of ecological, cultural and social determinants of health with different levels of impact. These underlying factors can also directly and indirectly hinder the effectiveness of MHBC interventions. Thus, understanding the interrelationships of barriers and facilitators along with their myriad drivers is essential in developing successful preventive health programs. Also, it is important to recognize that, to target successful and sustainable behavior changes in chronic disease risk factors, single-level approaches and interventions are not sufficient. Importantly, based on the findings from this systematically conducted narrative review, MHBC interventions targeting different intervention levels should be optimized by promoting authentic and effective collaboration of different health and educational sectors, organizations, and community members, with a strong emphasis on Indigenous participation and leadership at all stages of program development and implementation. Adding community-based interventions to target chronic disease risk behaviors could improve the outcome and complement the use of individual-based MHBC brief interventions among Indigenous Australian populations [18]. Lastly, findings from evidence-based evaluations of Indigenous-specific MHBC brief intervention programs in Australia should assist in informing policy and research in Canada and New Zealand.

## Supplementary information

**Additional file 1.** Search Subject Headings and Term Harvesting Table.

**Additional file 2.** PRISMA 2009 Checklist.

## Data Availability

Data accessed and analyzed during this review are available from the corresponding author on request.

## Abbreviations

AIHW: Australian Institute of Health and Welfare
AIQ: Adult Impact Questionnaire
BI: Brief intervention
BMI: Body mass index
HFN: Healthy Foods North
MCHW: Maori Community Health Worker
MHBC: Multiple health behavior change
SBIRT: Screening, Brief Intervention, and Referral to Treatment
SES: Socioeconomic status
SNAP: Smoking, nutrition, alcohol, and physical activity
SNP: Smoking, nutrition, and physical activity
WHO: World Health Organization

## References

[1] Glover M, Kira A, Johnston V, Walker N, Thomas D, Chang AB, Bullen C, Segan CJ, Brown N (2015). A systematic review of barriers and facilitators to participation in randomized controlled trials by indigenous people from New Zealand, Australia, Canada and the United States. Glob Health Promot.

[2] Campbell D, Pyett P, McCarthy L (2007). Community development interventions to improve aboriginal health: building an evidence base. Health Sociol Rev.

[3] Foulds HJ, Bredin SS, Warburton DE (2011). The effectiveness of community based physical activity interventions with aboriginal peoples. Prev Med.

[4] Utter J, Scragg R, Schaaf D, Fitzgerald E (2006). Nutrition and physical activity behaviours among Mäori, Pacific and NZ European children: identifying opportunities for population-based interventions. Aust N Z J Public Health.

[5] Sharma S (2010). Assessing diet and lifestyle in the Canadian Arctic Inuit and Inuvialuit to inform a nutrition and physical activity intervention programme. J Hum Nutr Diet.

[6] World Health Organization. Chronic diseases and health promotion. Part Two: the urgent need for action [Internet]: World Health Organization; 2015. Available from: https://www.who.int/chp/chronic_disease_report/part2_ch1/en/index12.html/. Accessed 22 June 2017

[7] Australian Institute of Health and Welfare (2010). Contribution of chronic disease to the gap in adult mortality between Aboriginal and Torres Strait Islander and other Australians. Catalogue No.: IHW 48.

[8] Noble N, Paul C, Sanson-Fisher R, Turon H, Turner N, Conigrave K (2016). Ready, set, go: a cross-sectional survey to understand priorities and preferences for multiple health behaviour change in a highly disadvantaged group. BMC Health Serv Res.

[9] Clifford A, Pulver LJ, Richmond R, Shakeshaft A, Ivers R (2010). Brief intervention resource kits for Indigenous Australians: generally evidence-based, but missing important components. Aust N Z J Public Health.

[10] Oosterveen E, Tzelepis F, Ashton L, Hutchesson MJ (2017). A systematic review of eHealth behavioral interventions targeting smoking, nutrition, alcohol, physical activity and/or obesity for young adults. Prev Med.

[11] Prochaska JJ, Prochaska JO (2011). A review of multiple health behavior change interventions for primary prevention. Am J Lifestyle Med.

[12] Panaretto K, Coutts J, Johnson L, Morgan A, Leon D, Hayman N (2010). Evaluating performance of and organisational capacity to deliver brief interventions in aboriginal and Torres Strait islander medical services. Aust N Z J Public Health.

[13] Werch C (2006). The behavior-image model: a paradigm for integrating prevention and health promotion in brief interventions. Health Educ Res.

[14] Armitage A. Xwi7xwa Collection. Comparing the policy of aboriginal assimilation: Australia, Canada, and New Zealand. Vancouver: UBC Press; BC. 1995.

[15] Gover K. Indigenous rights and governance in Canada, Australia, and New Zealand: Oxford University Press; 2011. https://www.oxfordbibliographies.com/view/document/obo-9780199756223/obo-9780199756223-0028.xml. Accessed 1 Nov 2018.

[16] B.strong [Internet]. B.strong. Available from: http://www.bstrong.org.au/ Accessed 12 Dec 2018.

[17] Nilsen P, Kaner E, Babor TF (2008). Brief intervention, three decades on: an overview of research findings and strategies for more widespread implementation. Nordic Stud Alcohol Drugs.

[18] Clifford A, Pulver LJ, Richmond R, Shakeshaft A, Ivers R (2011). Smoking, nutrition, alcohol and physical activity interventions targeting indigenous Australians: rigorous evaluations and new directions needed. Aust N Z J Public Health.

[19] Peters MD, Godfrey CM, McInerney P, Soares CB, Khalil H, Parker D. The Joanna Briggs institute Reviewers’ manual 2015: methodology for JBI scoping reviews. Adelaide: The Joanna Briggs Institute; 2015.

[20] Lockwood C, Porrit K, Munn Z, Rittenmeyer L, Salmond S, Bjerrum M, Loveday H, Carrier J, Stannard D. Chapter 2: systematic reviews of qualitative evidence. In: Aromataris E, Munn Z (Editors)*.* Joanna Briggs Institute reviewer’s manual. The Joanna Briggs Institute, 2017. Available from: https://reviewersmanual.joannabriggs.org/. Accessed 20 Dec 2018.

[21] Simmons D, Rush E, Crook N, Rona TW o, Diabetes Prevention Strategy Team (2008). Development and piloting of a community health worker-based intervention for the prevention of diabetes among New Zealand Maori in Te Wai o Rona: diabetes prevention strategy. Public Health Nutr.

[22] Coppell KJ, Tipene-Leach DC, Pahau HL, Williams SM, Abel S, Iles M, Hindmarsh JH, Mann JI (2009). Two-year results from a community-wide diabetes prevention intervention in a high risk indigenous community: the Ngati and healthy project. Diabetes Res Clin Pract.

[23] Hamerton H, Mercer C, Riini D, McPherson B, Morrison L (2012). Evaluating Māori community initiatives to promote healthy eating, healthy action. Health Promot Int.

[24] Tomlin D, Naylor PJ, McKay H, Zorzi A, Mitchell M, Panagiotopoulos C (2012). The impact of action schools! BC on the health of aboriginal children and youth living in rural and remote communities in British Columbia. Int J Circumpolar Health.

[25] Anand SS, Davis AD, Ahmed R, Jacobs R, Xie C, Hill A, Sowden J, Atkinson S, Blimkie C, Brouwers M, Morrison K (2007). A family-based intervention to promote healthy lifestyles in an aboriginal community in Canada. Can J Public Health.

[26] Mead EL, Gittelsohn J, Roache C, Corriveau A, Sharma S (2013). A community-based, environmental chronic disease prevention intervention to improve healthy eating psychosocial factors and behaviors in indigenous populations in the Canadian Arctic. Health Educ Behav.

[27] Tyler DO, Horner SD (2008). Collaborating with low-income families and their overweight children to improve weight-related behaviors: an intervention process evaluation. J Spec Pediatr Nurs.

[28] Towns C, Cooke M, Rysdale L, Wilk P (2014). Healthy weight interventions in aboriginal children and youth: a review of the literature. Can J Diet Pract Res.

[29] Kuninkaanniemi H, Villberg J, Vanhala M, Poskiparta M (2011). Behaviour-change interventions in primary care: influence on nutrition and on the metabolic syndrome definers. Int J Nurs Pract.

[30] Wilk P, Cooke M (2015). Collaborative public health system interventions for chronic disease prevention among urban aboriginal peoples. Int Indigenous Policy J.

[31] Gibson O, Lisy K, Davy C, Aromataris E, Kite E, Lockwood C, Riitano D, McBride K, Brown A (2015). Enablers and barriers to the implementation of primary health care interventions for indigenous people with chronic diseases: a systematic review. Implement Sci.

[32] Hardeman W, Johnston M, Johnston D, Bonetti D, Wareham N, Kinmonth AL (2002). Application of the theory of planned behaviour in behaviour change interventions: a systematic review. Psychol Health.

[33] Naylor PJ, Scott J, Drummond J, Bridgewater L, McKay HA, Panagiotopoulos C (2010). Implementing a whole school physical activity and healthy eating model in rural and remote first nations schools: a process evaluation of action schools! BC. Rural Remote Health.

