# Supplementary material for: Barriers and facilitators to the implementation of brief interventions targeting smoking, nutrition, and physical activity for indigenous populations: a narrative review
Source: Int J Equity Health. 2019 Nov 5;18:169. doi: 10.1186/s12939-019-1059-2 (PMC6833184; doi:10.1186/s12939-019-1059-2)
Supplement: Supplementary file 1 — Additional file 1. Search Subject Headings and Term Harvesting Table. [file 12939_2019_1059_MOESM1_ESM.docx]

**Additional file 1: Search Subject Headings and Term Harvesting Table:**

| **Database** | **1. Brief Intervention** | **2. Areas of Risk: Poor nutrition, physical activity, and smoking** | **3. Indigenous Populations** |
| --- | --- | --- | --- |
| **PubMed** | brief[All Fields] AND ("Intervention (Amstelveen)"[Journal] OR "intervention"[All Fields] OR "Interv Sch Clin"[Journal] OR "intervention"[All Fields]) AND "smoking cessation"[MeSH] OR  “Early Medical Intervention” [MeSH] | “Nutrition Therapy” [MeSH] OR “Nutrition Assessment” [MeSH] OR “ Nutrition Surveys’ [MeSH] OR “Diet, food, & nutrition’ [MeSH] OR “Diet Therapy” [MeSH]  “smoking cessation” [MeSH] OR  “Exercise” [MeSH] | “Indians, North American” [MeSH] OR “Population Groups” [MeSH]  ((((((Indigenous[Text Word] OR Aboriginal*[Text Word] OR "first nations"[Text Word] OR inuit*[Text Word] OR metis [Text Word] OR indian[Text Word] OR "Torres strait islander*"[Text Word] OR maori[Text Word] OR "native american"[Text Word] OR eskimo[Text Word]))) OR ((“Indians, North American” [MeSH])))) |
| **Embase** | (brief intervention or brief interventions or SBIRT).mp. [mp=title, abstract, heading word, drug trade name, original title, device manufacturer, drug manufacturer, device trade name, keyword, floating subheading word] | (nutrition or smoking or physical activity or physical or SNAP) | (Indigenous or Aborigin* or first nations or inuit* or metis or native or indian or "aboriginal and Torres strait islander" or maori or eskimo*).mp. |
| **CINAHL** | Brief intervention or screening  or motivational or SBIRT | (MH "Nutrition") OR (MH "Diet+") OR (MH "Nutritional Status") OR (MH "Nutritional Requirements+") OR (MH "Nutritional Assessment") OR (MH "Smoking Cessation Programs") OR (MH "Smoking+") OR (MH "Tobacco Use Cessation Products+") OR (MH "Exercise+") OR (MH "Physical Fitness+") OR (MH "Physical Activity") | (MH "Indigenous Peoples+") |
| **HealthStar** | (brief intervention or brief interventions or SBIRT).mp. [mp=title, original title, abstract, name of substance word, subject heading word] | Nutrition Assessment/ or exp Nutrition Surveys/ or diet/ or exp food habits/ or exp nutritional requirements/ or nutritional status/ or exp "Tobacco Use"/ or exp "Tobacco Use Cessation"/ or exp Exercise/ or Physical Fitness/ or exp Overweight/ | (((Indigenous or Aboriginals or first nations or inuits or metis * or native or indian or aboriginal) and Torres strait islander) or maori).mp. [mp=title, original title, abstract, name of substance word, subject heading word] |
| **PsycINFO** | SU.EXACT("Intervention")  OR SU.EXACT("Brief Psychotherapy")  OR  (DE "Intervention" OR DE "Behavior Change") AND (DE "Behavior Therapy" OR DE "Behavior Change" OR DE "Motivational Interviewing" OR DE "Health Behavior" | SU.EXACT("Fast Food") OR SU.EXACT("Diets") OR SU.EXACT("Eating Behavior") OR SU.EXACT("Nutrition") OR SU.EXACT.EXPLODE("Nutrition") OR SU.EXACT.EXPLODE("Overweight") OR SU.EXACT("Weight Control") OR SU.EXACT.EXPLODE("Tobacco Smoking") OR SU.EXACT("Nicotine Withdrawal") OR SU.EXACT("Smoking Cessation") OR SU.EXACT.EXPLODE("Physical Activity") OR SU.EXACT("Physical Fitness") | (DE "Indigenous Populations")  SU.EXACT.EXPLODE("Indigenous Populations") |
| **Web of Science** | TS=(Brief intervention* or screening or motivation* or SBIRT | NA | TS=( Indigenous OR Aboriginals or first nations OR inuits OR metis * OR native OR indian OR aboriginal and Torres strait islander OR maori ) |
| **Keywords** | Brief intervention* OR  Brief intervention program* OR  Brief intervention training program* OR brief motivational intervention* OR brief advice* OR motivational interviewing* OR SBIRT* | Nutrition * OR Nutritional * OR * Diet * OR Food * OR Dietary *  Smoking * OR tobacco *  Physical activity * OR exercise *  Physical activity* OR Exercise* OR Physical exercise* | Aboriginals or first nations OR inuit(s) OR metis* OR native OR indian OR aboriginal and Torres strait islander OR maori OR eskimo* |
